# Editorial: The effects of COVID-19 on cancer research methods & strategies

**DOI:** 10.3389/fpubh.2022.988736

**Published:** 2022-08-10

**Authors:** Louis Fox, Richard Sullivan, Deborah Mukherji, Mieke Van Hemelrijck

**Affiliations:** ^1^King's College London, London, United Kingdom; ^2^Lebanese American University, Beirut, Lebanon

**Keywords:** COVID-19, cancer, research, methods, recruitment

The ongoing SARS-CoV-2 pandemic continues to inflict a severe public health challenge throughout the world (1). The ease *via* which the SARS-CoV-2 virus is transmitted, the rapidly evolving variants, combined with the potential for cases to require acute care and the impact of non-pharmaceutical interventions (e.g., lockdowns), has meant that healthcare systems have been significantly disrupted by the outbreak (2).

It is evident that the provision of cancer services and patient outcomes has been strongly impacted by the outbreak of SARS-CoV-2 (3–5). Suspected cancer referrals have declined sharply, creating a concern that many individuals could be missing an opportunity for curative treatment (diagnostic delays) (6, 7). Furthermore, consistent clinical guidelines are yet to be established for the management of cancer patients in a SARS-CoV-2 epidemic and the friction within pathways of care has dramatically increased (changes to treatment and treatment delays). These circumstances need to be considered in the context of multi-center research suggesting that cancer-related risk factors such as hematologic malignancy and recent cytotoxic therapy are associated with increased mortality in COVID-19-positive patients (8); and increased breakthrough infections of COVID-19 despite vaccination (9). The latter finding is especially pertinent to any public health policy that leans heavily into vaccination programmes as opposed to non-pharmaceutical interventions, as is now increasingly the case.

Whilst work to address issues related to the immediate provision of cancer care is extremely urgent, it is also important to examine the impact that the pandemic may be having on cancer research more broadly (10). Governmental agencies have been issuing guidance regarding clinical trials during the pandemic, and research teams have been adapting, with some reporting successful clinical trials continuity. Furthermore, the arrival of comprehensive data resources such as National COVID Cohort Collaborative (N3C) Data Enclave (8, 9) will assist in the construction of informed guidelines for cancer management going forward. However, there remain important questions with regards to the impact that the COVID-19 pandemic may be having on the future of cancer research methods and strategies, globally. The pandemic has and continues to have very different impacts on countries' ecosystems.

This Research Topic specifically focused on the following areas: impact of COVID-19 on the conduct of cancer research both from a practical as well as behavioral or sociological point of view; impact of COVID-19 on cancer research strategies and priorities, taking into account the economic impact on federal and philanthropic organizations.

The work by Murillo et al. evaluated this specifically for Colombia, a country where cancer research was already suboptimal prior to the COVID-19 pandemic. By searching Web of Science as well as local and national gray literature, combined with interviewing principal investigators, they explored the impact on research funding, output, and conduct. Whilst a decline was observed at national level, at institutional level research remained fairly stable – likely due to the predominance of observational studies. However, alternatives to ensure research continuity have been scarcely implemented given the limited access and low technology in this middle-income country.

These observations are in line with a literature review conducted by our own team to qualitatively examine the documented impacts of the pandemic on cancer researchers (Fox et al.). A total of 215 articles were subjected to a conventional qualitative content analysis and showed extensive practical and economic effects on the field of cancer research: 1) COVID measures halting cancer research activity entirely; 2) COVID measures limiting cancer research activity; 3) forced adaptation of research protocols; 4) impacts on cancer diagnosis, cases, and services; 5) availability of resources for cancer research; 6) disruption to the private sector; and 7) disruption to supply chains. Three categories of consequences from these impacts also emerged: 1) potential changes to future research practice; 2) delays to the progression of the field; and 3) potential new areas of research interest.

Apart from the practical and economic consequences, it also needs to be noted that staff and patients in the context of cancer research are affected by the pandemic. The Belgian SocieTy for Radiation Oncology (BeSTro) sent weekly national survey sent to their radiotherapy departments to evaluate impact on clinical management and research activities (Vaandering et al.). A drop in patients treated was noted over time, however treatment was continued in many of the COVID-positive patients with fractionation schemes being adapted. The latter highlights that flexibility around guidelines and management is key to ensuring continuation of treatment and research activities.

However, patients have not only been affected in the context of treatment, but also research activities. We interviewed 13 participants from the UK who were purposively sampled, including a broad sample of cancer types, and a mixture of individuals who have and have not taken part in research previously (Fox et al.). Our findings indicated that cancer patient decision-making about research participation during an infectious disease pandemic was consistent with basic cost-benefit models of decision-making. The provision of practical solutions that can be considered at the study protocol design stage, such as safe travel, information, and the use of staff and study sites familiar to the patient, can seemingly influence this decision-making by reducing anxiety about infection risks and hence support patients' inherent motivation to do something for the good of wider society.

As such, it is also important to have the right validated research tools to measure the cancer patient experience in the context of COVID-19. Ahn et al. therefore investigated how useful the six-item Stress and Anxiety to Viral Epidemics (SAVE-6) scale and the Coronavirus Anxiety Scale (CAS) are as tools to assess anxiety related to coronavirus disease (COVID-19) in cancer patients. Based on a sample of 221 patients, it was found that the SAVE-6 and CAS could be used to evaluate moderate and severe degrees of functional impairment related to mental health, respectively, in cancer patients during viral epidemics.

In conclusion, we would like to stress the extensive practical and economic effects of the COVID-19 pandemic on the field of cancer research. Assessment of cancer research strategies in a post-COVID world should acknowledge this potential for substantial limitations (including budget, access to patients, staffing, or supply chain issues), whilst keeping in mind exacerbated cancer disparities, advances in digital health, and new areas of research related to the intersection of cancer and COVID-19. The studies presented in this Research Topic point to ways in which the situation in cancer research could be alleviated. Management of cancer patients can be made flexible, when combined with surveillance of real-world evidence that can provide confidence that patient care and outcomes are not being detrimentally affected (Vaandering et al.). Clinical trials can be designed with patient accessibility in mind, clear information about COVID-19 precautions to give confidence to participants (Fox et al.), and dynamic research protocols which shed the rigidities that can make studies vulnerable to disruption (Fox et al.). Funding resources should be mobilized and strategically targeted, to protect the vulnerable via vaccination research and continuity of freely available COVID-19 testing; and to protect the overall continuity of scientific progress which could save many lives. Strategic allocation of resources can help low- and middle-income countries to not be disadvantaged in research prioritization; and could maintain annual influxes of fresh graduate talent. We are starting to have good knowledge of the problems – now it is time for solutions.

## Author contributions

MV and LF drafted the editorial. RS and DM critically revised. All authors contributed to the article and approved the submitted version.

## Conflict of interest

The authors declare that the research was conducted in the absence of any commercial or financial relationships that could be construed as a potential conflict of interest.

## Publisher's note

All claims expressed in this article are solely those of the authors and do not necessarily represent those of their affiliated organizations, or those of the publisher, the editors and the reviewers. Any product that may be evaluated in this article, or claim that may be made by its manufacturer, is not guaranteed or endorsed by the publisher.
